# The Diversity of Bacterial Lifestyles Hampers Bacteriophage Tenacity

**DOI:** 10.3390/v10060327

**Published:** 2018-06-15

**Authors:** Marta Lourenço, Luisa De Sordi, Laurent Debarbieux

**Affiliations:** 1 Department of Microbiology, Institut Pasteur, F-75015 Paris, France; marta.mansos-lourenco@pasteur.fr (M.L.); luisa.de-sordi@pasteur.fr (L.D.S.); 2 Collège Doctoral, Sorbonne Université, F-75005 Paris, France

**Keywords:** virus–host interactions, bacteriophage efficacy, gastrointestinal tract, phage therapy

## Abstract

Phage therapy is based on a simple concept: the use of a virus (bacteriophage) that is capable of killing specific pathogenic bacteria to treat bacterial infections. Since the pioneering work of Félix d’Herelle, bacteriophages (phages) isolated in vitro have been shown to be of therapeutic value. Over decades of study, a large number of rather complex mechanisms that are used by phages to hijack bacterial resources and to produce their progeny have been deciphered. While these mechanisms have been identified and have been studied under optimal conditions in vitro, much less is known about the requirements for successful viral infections in relevant natural conditions. This is particularly true in the context of phage therapy. Here, we highlight the parameters affecting phage replication in both in vitro and in vivo environments, focusing, in particular, on the mammalian digestive tract. We propose avenues for increasing the knowledge-guided implementation of phages as therapeutic tools.

## 1. Introduction

With the alarming worldwide increase in the prevalence of multidrug-resistant bacteria, phage therapy—the use of phages to target pathogenic bacteria [1]—has recently returned to the spotlight in the USA and Europe, although it had never fallen out of favour in countries such as Georgia [2]. The three main characteristics of phages that make phage therapy an appealing strategy are (i) the self-replication of phages, leading to a local increase in their concentration; (ii) the lack of broad off-target effects due to the narrow host specificity of phages and (iii) genomic flexibility making it possible to rapidly develop optimised variants. The recent publication of a successful compassionate clinical case treatment with phages has highlighted the potential value of phage therapy in the context of human health [3,4]. However, in modern phase II clinical trials, the efficacy of phage therapy was highly variable in a small number of patients with chronic otitis, and phage therapy was ineffective in a larger trial with children with diarrhoea [5,6]. This lack of success may partly reflect the paucity of data relating to the translation from in vitro to clinical settings [7]. We must, therefore, address the challenge of identifying the parameters characterising effective phage treatments. For example, in studies of several experimental models investigating the use of phages to target bacteria residing in the digestive tract of animals, treatment efficacy has been reported to range from complete inefficacy to highly successful [8,9,10,11,12]. These findings contrast strongly with in vitro observations in which most, if not all, phages are highly efficient at infecting their host. These discrepancies may be explained by the influence of the bacterial lifestyle on phage infection, as discussed below.

## 2. Bacteria Provide Essential Support for the Parasitic Lifestyle of Phages

Bacteria are among the most ubiquitous organisms on the planet and their high levels of diversity are regularly confirmed in metagenomics studies [13,14,15]. Bacteria colonise a multitude of environments, from oceans to deserts, demonstrating their great ability to thrive in different environments and to regulate major global processes, such as the biogeochemical cycles of essential elements (carbon, nitrogen, oxygen) [16].

From an anthropocentric point of view, most bacteria are harmless while a few are beneficial or pathogenic. Bacteria isolated from many body sites have been shown to survive in various conditions, such as the acidic medium of the stomach or the highly oxygenated respiratory tract. Even within a single species, bacteria may display considerable phenotypic flexibility. This is illustrated by the well-known model bacterium *Escherichia coli*, a facultative anaerobe able to survive in environmental conditions that are very different from its natural habitat, the digestive tract of warm-blooded animals [17].

Bacterial physiological responses play a crucial role in shaping the interactions of bacteria with their environment. The recent development of several techniques (membrane, chip, RNASeq), which facilitate the capture of mRNAs, has made a fundamental contribution to the description of global physiological responses in bacteria. These techniques have made it possible for researchers to describe the transcriptomic profile of bacteria growing in several different types of conditions [18,19,20,21,22,23]. For example, Denou et al. compared *Lactobacillus johnsonii* gene expression between in vitro (in flasks) and in vivo (mouse gastrointestinal tract) conditions and in different sections of the gastrointestinal tract (stomach, caecum and colon) [18]. Their observations confirmed that the animal host, either directly or indirectly via other microbes, influences gene expression in the bacterial populations colonizing different body sites.

**Figure 1:**
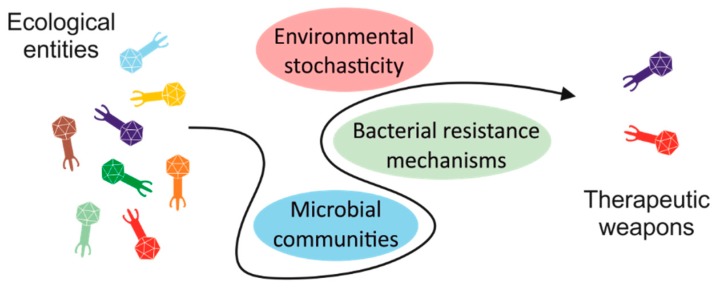
Schematic illustration summarising the obstacles that bacteriophages must overcome to be considered as antibacterial weapons.

Phages are obligate parasites and, as such, their distribution matches that of the bacteria they infect. Bacteria may be susceptible to phages or resistant via many mechanisms developed by bacteria during the course of their coevolution with phages. Bacteria can prevent phage adsorption by deleting phage receptors, modifying their conformation, or releasing factors that occupy the binding site or even mask it. Other mechanisms of protection involve the prevention of phage DNA injection, the digestion of phage DNA by restriction-modification enzymes or by the CRISPR-Cas machinery. For a more comprehensive and detailed description of these phage resistance mechanisms, we refer the reader to the review by Labrie, S.J., et al. [24]. In 2015, a novel system called BREX (bacteriophage exclusion) was described and reported to specifically prevent phage DNA replication [25]. Doron et al. (2018) recently used comparative genomics to predict an impressive list of 26 new putative antiphage systems, nine of which were experimentally validated [26]. In addition, environmental fluctuations driving bacterial modifications can directly or indirectly influence phage infection, as discussed in the chapters below focused on virulent phages and schematically illustrated in Figure 1.

## 3. Bacterial Physiology Affects the Outcome of Phage Infection

In optimal in vitro conditions, bacterial growth is characterised by four different phases: (i) the lag phase (initial phase) during which the bacteria are still adapting and adjusting to the growth conditions; (ii) the exponential growth or log phase during which the bacteria replicate rapidly; (iii) the stationary phase during which nutrients are depleted from the medium, limiting replication rates (during this phase, growth rate and death rate are usually matched); and (iv) death, which occurs when the nutrients are exhausted. The physiological state of a bacterium is linked to its growth conditions, which are, in turn, highly dependent on abiotic factors, such as nutrient variety and density, in particular [19]. Changes in growth conditions can affect the antibacterial activity of phages by preventing infection, replication or lysis. In vitro studies of phage–host interactions are typically performed in exponential phase cultures in liquid broth and little is known about these interactions in other conditions resembling those found in natural environments. The initial isolation of phages itself introduces a selection bias in that it often occurs in growth conditions that are optimal for the host (rich medium with shaking), i.e., those in which the bacteria are constantly in a planktonic state.

Many in vitro studies on the model system consisting of the phage T4 and its host, *E. coli*, have characterised the effects of host physiology on the infection efficiency of the phage. At high growth rates, phage T4 is absorbed and released more rapidly, its burst size increases and its eclipse and latent periods decrease [27,28,29,30]. These observations led to the suggestion that phage synthesis and assembly rates depend on the protein synthesis machinery of the host, whereas lysis time is correlated with cellular dimensions [29]. Other studies have shown that phages T4 and ms2 can enter a dormant state during the infection of stationary-phase cells. This state has been referred to as “hibernation” and is reversible. Some phage proteins are synthesised during hibernation but particle assembly is placed on hold until additional nutrients become available in the environment, which allows the phage infection processes to resume [27,31,32].

Bacteria may display various physiological states due to environmental stochasticity, which can convert a phage-susceptible bacterial host into a phage-resistant host. Indeed, stochastic differential gene expression can generate a heterogeneous population of cells within which a subpopulation may express lower levels of phage receptors, with consequences for the rate of phage adsorption. Such stochastic expression renders cells effectively resistant to phages without the need to acquire resistance through mutation. Although this phenomenon, known as phenotypic resistance, remains underappreciated and understudied, it may potentially account for the difference in infection efficiency between in vitro and in vivo conditions [33,34,35].

Another example of differences in phage infection efficiency due to shifts of environmental conditions is provided by phage T5. The infection efficiency of this phage has been shown to be dependent on temperature, which alters the host cell’s membrane rigidity [36]. By contrast, *E. coli* phage infection efficiency seems to be independent of oxygen concentration, at least in vitro, as shown by studies in both aerobic and anaerobic conditions [11,12]. Nevertheless, it was shown that different aeration conditions imposed on *Bacillus thuringiensis* could affect the duration of the infectious cycle of phage BAM35 [37]. In 2004, Sillankorva et al. performed an extensive study with the phage US1 and its host, *Pseudomonas fluorescens* [38]. These authors showed that temperatures lower (4 °C) or higher (37 °C) than the optimal temperature (26 °C) had a major effect on phage infection efficiency, leading to an absence of phage amplification (37 °C) or rare (4 °C) phage infections. Furthermore, this phage cannot infect its host in a glucose medium despite its high infection efficiency in nutrient-rich conditions. Studies of the outer membrane protein profiles of cells grown in these two environments identified two proteins—17.5 and 99.0 kDa—with differential abundance under these growth conditions. These proteins were not detected in bacteria growing at 37 °C or in a glucose medium and the smaller protein was not detected at 4 °C, suggesting a possible role for these proteins as phage receptors. Environmental shifts can also, in some cases, trigger the production of capsules, which may mask phage receptors or allow other phages to use these same receptors [39,40,41]. In other cases, these environmental fluctuations can promote the induction (resumption of lytic cycle) of prophages present in the genome of bacteria, causing the destruction of their host [42]. Interestingly, prophage induction is frequent in the digestive tract of mammals as suggested by metagenomics data, however, their precise role waits to be defined [43,44].

## 4. Bacterial Community Lifestyle Influences Phage Infection

In any environment, including body sites, bacterial populations do not generally adopt the planktonic state of growth that is frequently observed in laboratory experiments. Instead, they tend to live in multilayer aggregates of cells that adhere to each other and frequently to surfaces via the production of a matrix of extracellular polymeric substances (EPSs) [45]. These EPSs include exopolysaccharides and proteins but also lipids and DNA. The resulting biofilms limit the efficacy of antibiotics, principally by decreasing their diffusion. As a result, the bacteria are not completely eradicated by such treatments, favouring the development of chronic bacterial infections [46]. In such situations, phages may constitute a potential solution given their impact on microbial communities [47]. However, the efficacy of phages against biofilms in vitro is variable and certain biofilm components may act as barriers against phage infection. For example, the presence of an amyloid fibre network of CsgA (curli polymer) can physically prevent phages from penetrating biofilms [48]. Phages can also attach to these amyloid fibres, preventing the viral binding to receptors [48]. On the other hand, some phages are equipped with enzymes that can degrade the polysaccharides produced by bacteria, thereby facilitating the diffusion of viral particles in biofilms [49,50]. The efficacy with which phages infect bacteria in biofilms is also strongly influenced by nutrient availability and nutrient concentrations that are highly heterogeneous within the biofilm structure [51].

An additional layer of complexity in interactions between phages and biofilms has been reported in studies of biofilms formed by the gut pathogen *Campylobacter jejuni*. Following phage infection, some of the cells in *C. jejuni* biofilms enter a carrier state. This involves phenotypic modifications to the bacterial cells, conferring advantages that enable them to survive in extraintestinal environments but preventing them from colonising the gut of chickens. Nevertheless, such carrier bacteria can import the phage into chickens that are already colonized by *C. jejuni*, providing the phage with opportunities to infect new cells following its release from the carrier [52,53].

Biofilms can also provide bacteria with a spatial refuge, reducing the probability of contact between a phage and its host, driving coexistence dynamics between the two populations without extinction of either the bacteria or the phage. This has been studied in vitro and modelled in silico. Spatially explicit individual-based stochastic models have shown that these structured refuges may maintain coexistence between the two populations within their boundaries, without the emergence of resistant clones [54]. In vitro experiments on populations of *P. aeruginosa* and bacteriophage PP7 in a heterogeneous artificial environment (static bacterial growth) showed a decrease in viral transmission and the emergence of refuges for the bacterial cells, stabilising interactions between the two antagonistic entities [55]. Similar observations were made when biofilms were grown on the wall of chemostats [56]. Finally, Eriksen et al. showed in a much more structured environment (solid agar in a Petri dish) that populations of phages and bacteria can co-exist in the long term but that this phenomenon is dependent on bacterial density, requiring the presence of at least 50,000 cells [57]. This threshold for phage replication is close to the threshold of 10,000 cells previously determined for well-mixed populations in several systems (*Bacillus subtilis*, *Escherichia coli* and *Staphylococcus aureus*), a phenomenon known as the “threshold for phage replication” or “proliferation threshold” [58,59].

## 5. Human Health and the Gut Phageome

Many aspects of phage biology, from initial adsorption to final lysis, can be affected by host behaviour, making it harder to reliably predict the overall efficacy of a phage in a given situation. This challenge is even greater when the complexity of viral species inhabiting the human gut is taken into account, as the cellular hosts of most of these viruses have yet to be identified [60,61].

The human gastrointestinal tract is a highly diverse and heterogeneous environment [62] that is inhabited by many different microorganisms [63]. It is also characterised by changes in conditions between sections, exposing its inhabitants to fluctuations in pH, nutrient levels, water and oxygen concentrations and even structure (ranging from liquid to semi-solid) [64,65,66,67,68].

It is now acknowledged that there are at least as many phages as bacterial cells in the mammalian gastrointestinal tract [69]. In healthy humans, only a small proportion of the phageome (phage community) is common to large numbers of individuals, with most of the phages present being subject specific [44]. Moreover, patients with inflammatory bowel disease (ulcerative colitis and Crohn’s disease) or AIDS have been shown to have gut viral populations that are very different in size and diversity from those of healthy individuals [70,71]. Furthermore, changes in viral diversity have been shown to precede the appearance of type I diabetes in children [72]. Phageome variations are of course connected with bacteriome deviations, demonstrating the intimate but still poorly characterised link between these two antagonistic populations. These conditions of viral and cellular dysbiosis raise questions about whether certain diseases are caused by changes in the microbiome rather than a single pathogen, defining the new concept of a “pathobiome” [73]. This concept underlies a paradigm shift with a move away from targeting single pathogens to targeting whole communities. Within this framework, phages are potentially useful as modulators of the microbiome as a whole. A striking example of this approach is provided by the similar efficacies of treatments for recurrent *Clostridium difficile* infections based on faecal microbiota transfer or sterile faecal transfer with filtering to exclude bacteria (but not phages), highlighting the role of non-bacterial components of the microbiota in the clinical effect of treatment [74,75]. Interestingly, the virome composition of patients treated by sterile transfer was found to be similar to that in the donor [75].

Interesting features of these phages can be linked to their adaptation to this environment; for example, some phages carry specific motifs in their capsids that allow them to bind to the intestinal mucus, potentially creating an additional layer of protection against bacteria [76]. Moreover, a direct role of the microbiome in phage evolution has also been suggested by the results of a study reporting the evolution of an ability to infect new hosts through the use of a second strain as a stepping stone [9]. No such evolution was observed in vitro or in dixenic mice and it was, therefore, suggested that the gut microbiota can promote phage and bacterial population diversification [9,77].

In summary, each partner in this tripartite interaction (the phage, the bacterium and the mammalian host) plays an important role in phage–bacterium dynamics. It is therefore vital to consider these partners as an ecosystem rather than as two separate paired entities (phage/bacterium or bacterium/host) [78,79]. There are currently gaps in our knowledge that we need to overcome if we are to implement effective strategies based on phage treatments for intestinal pathogens or for the development of microbiota engineering strategies.

## 6. Overcoming the Limitations of Phage Infection Efficacy In Vivo

To optimise the output of applications based on phages, the gap between in vitro studies and in vivo conditions may be bridged in several ways. First, phages can be isolated and characterised in more realistic and ecologically relevant conditions than under the conditions for optimal bacterial growth that are typically used. For example, we can decide to start from in vitro biofilms consisting of single bacterial species or multi-species communities, and then proceed to ex-vivo conditions using organs [11,80] and, ultimately, in vivo environments [60]. Second, the precise identification of phage receptors and their expression profiles in ecologically relevant conditions will not only provide us with information about phage biology but will also guide the optimisation of conditions for in vivo efficacy. Adaptation of the phage to the targeted pathogen has also been shown to increase phage efficacy in some cases [81]. Moreover, the use of different doses and the localised release of microencapsulated phages may overcome some of the difficulties related to bacterial refuges and bacterial density thresholds [82].

Third, the use of phages together with other treatments (e.g., antibiotics) may improve overall treatment efficacy, an idea that has gained ground since the publication of the Phage Antibiotic Synergy system in 2007 [83]. Several studies have since confirmed the advantages of combining these two antibacterial weapons, although some of the mechanisms involved have yet to be identified (not all phage and antibiotic combinations display such synergy [84,85]). Such combinations may also be effective against biofilms, overcoming the limitations of each of these agents used separately [86,87,88]. The selection of resistant cells is a key concern in the use of both antibiotics and phages. However, there is no overall association between antibiotic resistance and phage resistance profiles supporting further their use in combination [89]. Nevertheless, double resistance or persister cells could provide a means for bacteria to protect themselves from these threats, however, this requires further studies. Interestingly, it was observed that the growth of phage-resistant bacteria during phage therapy in experimental models can be controlled with two independent allies: antibiotics, as demonstrated in an endocarditis model, and the innate immune response, as shown in a model of pulmonary infection [84,90].

About a century after their first use as an antibacterial agent for treating infections, phages have not yet revealed all their secrets. Phage biology is presenting scientists with new challenges every day. Many of the mechanisms involved in phage infection of bacteria remain unknown, hindering the effective use of phages as an ecological and sustainable alternative or complement to overcome the antibiotic resistance crisis and to tackle diseases caused by microbiome dysbiosis.
